# Spinal Anesthesia for Awake Spine Surgery: A Paradigm Shift for Enhanced Recovery after Surgery

**DOI:** 10.3390/jcm13175326

**Published:** 2024-09-09

**Authors:** John Preston Wilson, Bryce Bonin, Christian Quinones, Deepak Kumbhare, Bharat Guthikonda, Stanley Hoang

**Affiliations:** Department of Neurosurgery, Louisiana State University Health Sciences Center, Shreveport, LA 71103, USA; jpw002@lsuhs.edu (J.P.W.); bryce.bonin@lsuhs.edu (B.B.); cjq001@lsuhs.edu (C.Q.); deepak.kumbhare@lsuhs.edu (D.K.); bharat.guthikonda@lsuhs.edu (B.G.)

**Keywords:** awake spine surgery, awake surgery, enhanced recovery after surgery (ERAS), spine surgery, minimally invasive surgery

## Abstract

Awake surgery has been applied for various surgical procedures with positive outcomes; however, in neurosurgery, the technique has traditionally been reserved for cranial surgery. Awake surgery for the spine (ASFS) is an alternative to general anesthesia (GA). As early studies report promising results, ASFS is progressively gaining more interest from spine surgeons. The history defining the range of adverse events facing patients undergoing GA has been well described. Adverse reactions resulting from GA can include postoperative nausea and vomiting, hemodynamic instability and cardiac complications, acute kidney injury or renal insufficiency, atelectasis, pulmonary emboli, postoperative cognitive dysfunction, or malignant hyperthermia and other direct drug reactions. For this reason, many high-risk populations who have typically been poor candidates under classifications for GA could benefit from the many advantages of ASFS. This narrative review will discuss the significant historical components related to ASFS, pertinent mechanisms of action, protocol overview, and the current trajectory of spine surgery with ASFS.

## 1. Introduction

Awake surgery for the spine (ASFS) is an alternative to general anesthesia (GA). As early studies report promising results, ASFS is progressively gaining more interest from spine surgeons [1,2,3]. The benefits offered by ASFS over GA are rooted in the enhanced recovery experienced by patients, including shorter lengths of hospital stay (LOS), reduced need for pain medications after surgery, reduced complications, and more timely recovery rates for patients [4]. Although data availability is seemingly limited, recent studies indicate that ASFS could surpass GA as the leading technique for achieving and maintaining adequate anesthetic depth during spine procedures [2,3,5]. ASFS still faces hurdles in the early days of defining this method. Solutions are still needed to identify the ideal patients, specify the range of pathology for which this approach is applicable, and understand how data collected from these procedures can be used to compare outcomes [3,6,7]. Nevertheless, using current protocols and techniques, ASFS has been successfully employed for cervical, thoracic, and lumbar procedures [8,9,10,11,12].

Awake surgery has been applied for various surgical procedures with positive outcomes; however, in neurosurgery, the technique has traditionally been reserved for cranial surgery [13,14,15,16,17,18,19]. The introduction of a paradigm shift focusing on enhanced recovery after surgery (ERAS) by Henrik Kehlet in 1997 provided the means by which awake surgery would eventually be delivered [20]. The ERAS protocol underpins preoperative and perioperative management using multimodal interventions for improved postoperative outcomes and patient recovery [21]. Traditionally, the ERAS protocols (specifically awake surgery) for spine surgery were thought to be too difficult of an undertaking due to the complexity of procedures. However, the early introduction of ERAS concepts for spine procedures has shown promising results for lumbar surgery (microdiscectomy, instrumented fusion, and anterior approaches), cervical spine surgery, scoliosis correction, and spinal oncology [4,22,23,24,25,26,27,28].

The success demonstrated in these fast-tracked ERAS protocols for these procedures sourced the foundation for what would eventually encompass ASFS. Shorter hospital stays and reduced complication rates translate to lower healthcare costs and hospital resource utilization. Furthermore, the focus on minimally invasive techniques and enhanced recovery protocols often associated with ERAS contribute to identifying other areas of improvement. This manuscript explores the historical aspects leading to the inception of current ASFS techniques and encompasses a narrative review centered around intrathecal administration. Specifically, the mechanisms of action, protocol overview, and the current trajectory of ASFS using the intrathecal technique are discussed.

## 2. Historical Background

### 2.1. The Introduction of Intrathecal Administration

The utilization of ASFS techniques, while novel in its regular application, is not as unique as originally thought. Although the method continues to be developed and further optimized, the logic stems from an old history. On 16 August 1898, August Bier, a German surgeon, first performed spinal anesthesia techniques at the Royal Surgical Hospital of the University of Kiel [29,30]. Bier’s patient was scheduled for segmented resection of the left ankle due to tuberculosis. However, the patient had experienced poor outcomes under GA. This prompted Bier to suggest the administration of 15 mg of cocaine to the intrathecal space of the spinal cord to allow him enough time to operate. This technique was coined by Bier as “cocainization of the spinal cord”. During the procedure, which would inadvertently be the first instance of spinal anesthesia (SA), the patient was vigilant but voiced no complaints of pain. Using similar SA methods, Bier implemented this approach for five more patients who underwent lower extremity surgery. “Cocainization of the spinal cord”, while effective in mitigating the pain experienced by patients during surgery, left patients bedridden for days due to debilitating “post spinal headache”. This pitfall, among many others, would leave Bier doubtful about the long-standing uses of the technique until more adequate neuraxial blocking agents could be developed.

### 2.2. Potential for Regional Blockade in Spinal Procedures

There is debate surrounding who first successfully implemented SA from the same era. James Corning, a New York neurologist, published his findings in 1885 on the applications of cocaine for spinal anesthesia for achieving vasoconstrictive and anesthetic effects [31]. Corning’s approach differed from Bier’s in the sense that Corning investigated the subcutaneous injection rather than the intrathecal administration of cocaine. Through what Corning would refer to as the “chemical transection of the spinal cord”, he achieved a similar nerve blockade of motor and sensory pathways [32]. Surrounding vasculature would absorb cocaine administered between two spinous processes, resulting in subsequent vasoconstriction and anesthetic effects. Over the next decade, following Corning’s subcutaneous approach and leading up to Bier’s accurate SA attempt, advances were made to allow for direct administration of pharmacologic agents to the intrathecal space, one of which being Heinrich Quincke, who would later serve as one of Bier’s educators. Heinrich is credited with developing the spinal needle for lumbar puncture procedures [33]. During Heinrich’s search for relief of increased intracranial pressure (ICP), he hypothesized that releasing cerebrospinal fluid from the spinal column would relieve symptoms due to elevated ICP. W. Essex Wynter, another surgeon from that era, was less successful with his approach to the subarachnoid space using a small capillary tube [34]. Conversely, Quincke’s design resulted in a more rigid spinal needle. Quincke’s improved design allowed improved access to spinal levels and related pathology. This spinal needle designed by Quincke was developed to maintain the material strength of the needle while carefully working down towards the spinal cord and its associated pathology.

### 2.3. Modern Medicine and Awake Spine Surgery

The advent of more advanced imaging, improvements in local anesthetics, and ERAS surgical techniques have pushed ASFS forward. Minimally invasive endoscopic spine surgery was the first instance in modern medicine where awake patients coordinated with the surgeon to target the vertebral level containing related pathology [35,36,37]. It was in 2019 that a group implementing ASFS first reported successful outcomes in 10 patients treated with minimally invasive techniques for interbody fusion [38]. Their approach consisted of a multimodal protocol utilizing endoscopic techniques, percutaneous screws, and optimized anesthetic agents for improved postoperative recovery times. For this study, the patients were consciously sedated with a continuous infusion of propofol and ketamine. For improved postoperative recovery and long-acting analgesic control, the patients were also given liposomal bupivacaine while closing. In 2022, a meta-analysis was performed on studies reporting a comparison of outcomes with either SA or GA. Surgery data for patients from 12 studies comprised 2796 lumbar spine surgery patients. The patients’ perioperative outcomes were compared between those receiving GA (1382 patients) and those receiving SA (1414 patients) [3]. Their analysis noted that the anesthesia mandates were not as thorough as the other metrics compared. However, all patients in the SA category were administered some variation of intravenous anesthesia for induction and maintenance of anesthetic depth (light to moderate conscious sedation). None of the patients were given paralytics or distributed opioids postoperatively, as neither was needed for SA patients. The key findings from this 2022 study were founded on SA’s ability to reduce LOS, blood loss, intraoperative time, pain, postoperative nausea and vomiting, and overall intraoperative complications. A handful of surgeons claim to be the “first” to complete an ASFS successfully; nonetheless, as it relates to ASFS using SA, studies began to report on the topic starting in 2019. One recent study comparing this method of ASFS utilizing SA with traditional GA reported robust findings for ASFS over a two-year study period from 2020–2022 [2]. They performed a matched cohort study comparing ten patients undergoing spinal fusion with SA for ASFS, with ten patients also being treated with spinal fusion, but they were administered GA. Interestingly, the patients in the ASFS cohort demonstrated reduced LOS, decreased need for opioids, and shorter times to ambulation. Although the sample size was small, the methodology of ASFS is promising and will most likely improve with more data as more surgeons adopt the paradigm.

Throughout the evolution of ASFS, several notable breakthroughs stand out as milestones in shaping its current practice. One of the most significant advancements was using local and regional anesthesia, which allowed for complex spine surgeries without needing GA [39]. This breakthrough demonstrated that patients could remain comfortable and awake during procedures. Another pivotal development was the refinement of minimally invasive surgical techniques, which, when combined with ASFS protocols, significantly reduced operative trauma and improved recovery times [40]. In recent years, using augmented reality and robotic assistance has led to unprecedented precision and efficiency in awake spine surgery [10,12].

## 3. Anesthesia for Awake Spine Surgery

The pharmacological foundation of SA for ASFS hinges on the efficacy and characteristics of potential anesthetic protocols that may be considered. It has been recommended that when using regional blockades for SA, a multimodal analgesic protocol be incorporated to aid in improving disability scores and decreasing overall postoperative complications [41]. Anesthetic protocols can vary depending on the intended approach for any given procedure. Neuronal blockade for ASFS can be achieved using three main methods: SA (intrathecal administration), epidural anesthesia (EA) via continuous flow through the catheter, or local anesthesia [39,42,43,44,45,46]. Table 1 provides an overview of ERAS protocols implementing different aspects of ASFS protocols and their findings as a result of such strategies.

### 3.1. Epidural Administration (EA)

As a method for administering nerve-blocking agents, EA is the least favorable among other options. EA is administered continuously into the epidural space via a catheter throughout the operation. This treatment effectively mitigates pain by inhibiting the transmission of signals through nerve fibers in and around the spinal cord. Specifically, the effects of this neuronal blockade help spine surgery teams achieve adequate sensory and motor blockades and provide analgesic effects. EA is a viable option for pain management both during and after surgery. Compared to SA, EA typically requires larger dosage quantities and does not achieve the same quality of neuraxial blockade. For this reason, most instances of ASFS will employ the SA approach with accompanying regional anesthesia to achieve the desired level of conscious sedation while maximizing analgesic effects where possible.

EA is typically reserved for multimodal anesthetic or analgesic regimens with accompanying local anesthesia (LA) to achieve proper physiologic nerve blocking for spinal procedures [50]. Surgeons in favor of EA with LA protocols during ASFS advocate for the technique with evidence based on reduced hospital costs and risks associated with surgery [51]. This finding is further evidenced by studies investigating the impact of GA or EA with or without LA on patient-reported outcomes (PROs) and complications. One study investigating these metrics across 5269 patients (724 GA, 4465 LA) treated with percutaneous endoscopic lumbar discectomy (PLED) found more impressive short-term improvement scores for Visual Analog Scale back pain and the Oswestry Disability Index for patients in the EA with or without LA group [52]. 

However, EA as a choice over LA is still actively being investigated as limited studies present a focused range of applications. A study investigating EA (366 patients) or LA (4099 patients) for PLED found that EA could be a reliable alternative for LA [53]. The spine surgery community is actively determining which surgical settings dictate the use of EA over either LA or SA. Evidence has been reported on the infrequent use of EA for PLED and other procedures. However, there remains a lack of robust evidence presented with randomized controlled trials comparing the efficacy and outcome on quantifiable metrics such as LOS [54]. Specific types of procedures or patients with demographic backgrounds may be good surgical candidates for ASFS with EA, such as procedures requiring longer operation times than SA can provide. However, there is a gap to be filled regarding the efficacy of LA as the stand-alone means for achieving conscious sedation during ASFS.

### 3.2. Local Anesthesia (LA)

LA has a robust history in spine surgery ever since the inception of percutaneous endoscopic procedures in 1999 [37,48,55]. During this procedure, local anesthetic solutions consisting of medications such as lidocaine, ropivacaine, and saline were administered to patients layer by layer during the surgical approach. This is valuable to anesthetic protocols in either GA or ASFS clinical settings. Notably, recent studies have shown improved intraoperative satisfaction and decreased LOS and anesthesia-related adverse reactions following gradient administration of LA for ASFS [9,44,47,48].

However, the concept itself is not novel, as the first instance of LA for spinal surgery in healthy patients was implemented in 1926 to remove spinal tumors [56,57]. Additionally, regarding ASFS, orthopedic surgeons have a well-documented history of applying LA for their procedures. Orthopedic surgeons have utilized variations of this technique, such as wide-awake local anesthesia with no tourniquet (WALANT). WALANT has been regularly implemented for hand and upper extremity procedures [58,59,60]. Implementing LA to target the erector spinae plane (ESP), first performed successfully in 2016, led to the more commonly practiced technique in earlier ASFS protocols [61]. Regional anesthesia in the form of ESP or other similar nerve blocks have been successfully implemented for transforaminal lumbar interbody fusion (TLIF) and other relatively short decompressive surgeries [62,63].

### 3.3. Spinal Anesthesia with Intrathecal Administration

SA, commonly recognized as the current gold standard for ASFS, is administered via a single-shot delivery to the intrathecal space. It is known for maintaining high anesthetic quality and rapid onset of action compared to EA (<5 min) [44]. However, traditional SA techniques, or intrathecal administration, suffer from short procedure times without a multimodal approach (2–4 h). The absence of supportive management provided by mild conscious sedation can lead to unforeseen risks and has typically been suggested only for relatively short procedures compared to lengthy, complex spine procedures [63]. Combined administration of SA, with an accompanying local anesthetic administered to the erector spinae or thoracolumbar interfascial plane, improves postoperative recovery times and pain management and shortens length-of-stay [1,4,38,64].

This technique commonly employs a multimodal anesthetic approach to keep patients stable and comfortable during the approach. The first step when utilizing this technique typically begins with administering an anesthetic mix of bupivacaine (1.8 to 2.5 mL of 0.5%) with fentanyl (15–20 μg) via lumbar puncture [43]. Once patients have been brought into the operating room, the second stage of the technique is implemented using ultrasound guidance for thoracolumbar interfascial plane (TLIP) administration. During stage two, another mixture of bupivacaine (up to 40 mL at 0.25%) will be delivered to the planes between the subcutaneous tissues and the multifidus and longissimus muscles bilaterally. The transition from stage one to stage two must be optimized and tailored to each patient to utilize the entire length of time of the anesthetic effects offered with this technique. A typical range of time between intrathecal administration and the beginning of the TLIP block is 10–20 min. There will be a brief waiting period following these two steps while the medication settles. During the waiting period for testing of anesthetic depth, the patient may be positioned, prepped, and draped until the surgical staff are comfortable with the neuraxial blockade achieved. After waiting an adequate amount of time for the medication to take effect, surgeons can test neuraxial blockade by subcutaneous infiltration at the level of the intended surgical site. If there is no pain, surgery teams may proceed with confidence. However, if pain is persistent, surgeons must determine the directionality of anesthetic migration. A second dose is administered if there is no migration of medication or if it is spotty on initial testing. This approach, or a similar alternative, would be necessary when using SA as there would not be another opportunity to readminister neuraxial blockade once the procedure commences. An example algorithm for testing SA efficacy after the initial administration of the first dose can be found in recent studies and can help determine if another dose is needed [44].

### 3.4. Mechanisms

**Figure 1:**
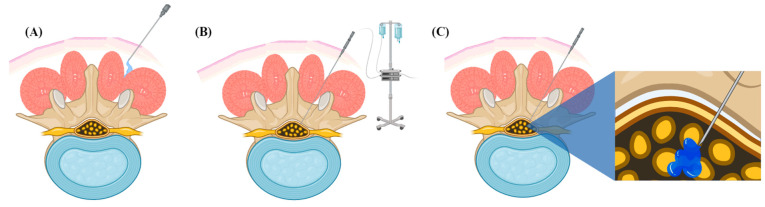
Methods of achieving sensory blockade in awake spine surgery. (**A**) Local anesthesia is delivered to the erector spinae plane to achieve temporary sensory blockade. (**B**) Epidural anesthesia is administered continuously into the epidural space via a catheter throughout the operation. (**C**) Intrathecal administration is administered as a single shot to the intrathecal space and provides the highest quality of sensory blockade for the most significant duration.

The mechanism by which medications used during intrathecal administration for ASFS exert their action is through the reversible blockade of voltage-gated sodium channels on the cytoplasmic cell surface, critical for action potential propagation along axonal cell membranes [65]. Choosing the right anesthetic agent involves consideration of several factors, such as the length of the surgery, the possible effects on the cardiovascular and neurological systems, and the patient’s medical history and allergies. Bupivacaine is often preferred for procedures requiring more prolonged periods of anesthesia, thanks to its extended duration (90–150 min) and fast onset of action (5–10 min) [65]. Additionally, bupivacaine has been actively investigated for its efficacy in ASFS protocols implementing an ESP approach. Conversely, lidocaine has a shorter onset of action (3–5 min) but suffers from a less impressive duration of neuraxial blockade (60–90 min). The pharmacokinetics of each agent, which encompasses its absorption, distribution, metabolism, and excretion, play a significant role in its clinical application. As such, it is essential to carefully consider and tailor the dosage and administration techniques to ensure optimal safety and efficacy. Figure 1 shows the different methods of anesthetic administration implemented during ASFS protocols.

## 4. Overview of the Application of Awake Spine Surgery

### 4.1. Patient Selection and Anesthesia Considerations

To discuss the application of ASFS, the following sections will be specific to SA with intrathecal administration as this technique is recognized as the most viable option compared to LA or EA. In applying SA for implementing ASFS, surgeons must first identify if a patient is a good candidate for awake surgery. Pediatric and elderly patients, as well as those with comorbidities such as hypertension, chronic obstructive pulmonary disease (COPD), or a long-standing history of tobacco use, will be poor surgical candidates for spine surgery under GA [66]. Nonetheless, these patients may still be potential favorable candidates for ASFS. For example, poor surgical candidates due to COPD or other chronic lung conditions may initially appear as potential ASFS patients. However, as seen with a COPD patient, ASFS procedures may introduce high risk to patients when operations mandate them to maintain their airways under mild sedation [67].

Nonetheless, regional anesthesia has demonstrated more favorable outcomes in the post-operative period than GA when administered to COPD patients [68]. These are new factors surgeons must consider when preparing for an ASFS procedure, especially considering that during unforeseen circumstances, primary troubleshooting will consist of transitioning the patient to endotracheal intubation and GA to maintain comfort during surgery and allow more time for surgical treatment. Generally speaking, patients treated with ASFS repeatedly show reduced LOS, time to recovery, and an overall increase in PROs [69].

Anesthesia has been known to cause an increase in adverse events during surgery for patients who have diabetes, heart disease, hypertension, kidney disease, lung disease, obesity, sleep apnea, or other neurological conditions (epilepsy or previous history of stroke) [70,71,72,73]. The range of adverse events affecting patients undergoing GA has been well described and is only expanding as our abilities to monitor patients improve. Well-known adverse events have been recognized as postoperative nausea and vomiting, hemodynamic instability and cardiac complications (arising from volatile anesthetics and medications like propofol), acute kidney injury or renal insufficiency, atelectasis, pulmonary emboli, postoperative cognitive dysfunction, or malignant hyperthermia and other direct drug reactions [44,72,74,75,76,77]. For this reason, many high-risk populations who have typically been poor candidates under classifications for GA could benefit from the many advantages of ASFS [1,70,74]. Other factors that favor ASFS include having less than two spinal levels needing treatment, the use of minimally invasive or endoscopic techniques, and elderly patient populations [19,63,64]. Factors that may contraindicate patients’ surgical candidacy for ASFS are high BMI, surgeries involving more than two spinal levels, bleeding disorders or coagulopathies, history of smoking, intracranial hypertension, demonstrating any qualities of respiratory compromise, unpredictable procedural time (primarily if more than 120 min), spinal stenosis above L2/3 (or other contraindications to SA), and stenosis that is too tight as it may prevent proper anesthetic spread due to alterations of CSF flow across the stenosis [43,44,76,77]. Once a patient has been identified as a good candidate for ASFS, providers may begin preliminary discussions about ASFS with patients and how this technique may be a better alternative for their surgical management.

### 4.2. Preoperative Stage

Following the patient’s consent to proceed with ASFS, preparations can be made with operating room staff on the day of surgery to allow the procedure to be performed as intended. Once the patient is admitted to preoperative holding, the first round of pre-emptive nonopioid analgesia medications can administered, including acetaminophen, gabapentin, pregabalin, dexamethasone, and NSAIDs [78,79,80]. Patients may feel anxious as the idea of ASFS can be pretty intimidating for both patients and surgical staff unfamiliar with the techniques [49,66]. Due to this, administering mild anxiolytics preoperatively may help relax the patient for surgery, as the patient’s belief in the procedure’s effectiveness is paramount for optimal recovery. Additionally, some surgery centers are implementing “intraoperative entertainment” forms, allowing patients to ease their minds during surgery and improve the overall patient experience [43]. Anesthesia teams may also perform an ESP block during operating room holding. This step, implemented into ASFS protocols, allows enough time before the incision for regional blockades to be fully functional by the time the incision begins. There is also the added benefit of opening up space in the operating room and improving surgical workflows by completing this step in the waiting bay.

### 4.3. Intraoperative Management

Following the patient entering the operating room, patients may position themselves on the surgical table while awake. Allowing the patient to position themselves on the surgical table improves patient positioning and comfort while decreasing the workload required to prepare the patient and room for surgery. Once the ESP block has been established, the room is prepared, and the patient is draped and ready for surgery. Infiltration with bupivacaine is performed to ensure adequate sensory blockade while approaching the intrathecal space. This step is often combined with either low-dose intravenous propofol or midazolam for light sedation. There has yet to be advice on using intraoperative neuromonitoring (IONM) during ASFS procedures as the technique is still being improved. A study investigating the changes in activation threshold for nerve irritation in the setting of SA found no differences in triggered-EMG (tEMG) thresholds between GA and SA patients [81]. The group’s conclusion suggests that once positive control values for tEMG are observed during SA for ASFS, similar to the observations under GA, the technique can be more regularly applied for ASFS procedures. Other considerations for intraoperative management during ASFS pertain to standard blood pressure support with phenylephrine or ephedrine, nausea prophylaxis with dexamethasone or ondansetron, and lastly, readministering bupivacaine after two hours in the setting of inadequate analgesia.

### 4.4. Postoperative Management

Postoperatively, patients are advised to avoid self-directed control of analgesia and manage in a standard fashion without traditional opioids for pain management when allowable. The intrathecal bupivacaine results in decreased overall opioid administration (if any at all) and lower rates of postoperative pain overall [5,82]. Postoperative nausea and vomiting may be controlled with ondansetron, and patients are encouraged to start ambulation with physical therapy on postoperative day 1. Table 2 shows the different protocol directives and goals with each stage of implementing an ASFS procedure under SA administration.

## 5. Pitfalls Associated with Awake Spine Surgery

Notably, one of the hurdles standing in front of the widespread adoption of ASFS techniques relates to patient hesitancy when being presented with awake surgery [49]. Undergoing a spine procedure, or any procedure for that matter, comes with a list of patient fears related to the nature of surgery. When presented with the choice of undergoing the operation with general anesthesia techniques or while being awake, the difficulties associated with choosing the awake protocol can be well understood. Future studies should continue to improve on these protocols and how they are presented to patients. Progress in awake spine surgery will be limited by patients’ perspectives on weighing the benefits of their postoperative care with the anxiety of being awake during the operation. Lastly, protocols used in ASFS need to be further improved to expand the range of procedures to which these techniques can be applied. The timeline allowed by ASFS techniques needs to be lengthened to perform complex spine procedures successfully.

## 6. Conclusions

ASFS has taken hold of the field of spine surgery as the next frontier for optimized patient outcomes and is quickly becoming the favored technique for achieving adequate sensory blockade for spinal procedures. The benefits offered by ASFS have been well defined in terms of improved recovery times, decreased LOS, and an overall reduction in surgery-related adverse events. For ASFS to progress further, additional improvements need to be made to surgical techniques, pharmacologic agents, and patient selection criteria. Future studies should examine the utilization of IONM in ASFS and gather more definitive data to create clinically acceptable protocols for use in operating rooms worldwide.

## Figures and Tables

**Table 1:** Modified anesthesia techniques implemented for Enhanced Recovery after Surgery and awake spine procedures.

Authors	Year of Publication	Summary
Yeung et al. [47]	2014	- A total of 30 patients were successfully treated with Endoscopic Foraminal Decompression under local anesthesia. - Complications included dysesthesia in 4 patients (resolved spontaneously in 3 patients within 2 months). - Three patients accepted and received fusion as a delayed staged procedure for residual back pain.
Wang et al. [38]	2016	- Ten patients underwent endoscopic interbody fusion combined with percutaneous screw fixation without general anesthesia and intubation. - The patients were surgically treated while under light sedation with continuous propofol and ketamine infusion. - Liposomal bupivacaine was injected into the posterior musculature using tracts made by inserting posterior pedicle screws. - There were no intraoperative or postoperative complications.
Kolcun et al. [5]	2019	- A total of 100 patients were treated with endoscopic transforaminal lumbar interbody fusion without general anesthesia. - Patients were kept under light sedation without intubation or use of narcotic agents, while liposomal bupivacaine was used for local analgesia. - Complications included four patient deaths unrelated to the surgery, two cases of cage migration, one case of osteomyelitis, and one case of endplate fracture.
Feng et al. [48]	2020	- A total of 50 patients underwent percutaneous endoscopic lumbar decompressive surgery using gradient local anesthesia of different concentrations injected into different tissues inside and outside the ligamentum flavum. - No serious complications were reported; however, three patients experienced transient postoperative dysesthesia of the lower limb (resolved spontaneously in 24 h).
De Biase et al. [11]	2021	- A total of 10 patients received transforaminal lumbar interbody fusion using a pedicle-based retraction system utilizing only regional anesthesia. - Intrathecal administration of bupivacaine with or without intrathecal narcotic (fentanyl or hydromorphone) was implemented with continuous sedative infusion for patient comfort (e.g., dexmedetomidine, propofol, midazolam, and fentanyl). - No complications were reported.
De Biase et al. [10]	2021	- Awake robotic transforaminal lumbar interbody fusion was performed on a patient using a surgical robot guidance system. - Given the patient previously experienced poor outcomes after administration of general anesthesia, they elected for awake surgery. - The patient was successfully treated with the operation with high patient satisfaction reported.
Azad et al. [49]	2022	- The outcomes of 301,521 patients who underwent cervical or lumbar spine operations with general anesthesia (294,903) or regional, epidural, spinal, and continuous sedation infusion (6618) were compared. - Analysis of cervical and lumbar procedures showed 30-day decreases in complication rates, readmission rates, and mean length of stay in the non-general anesthesia cohort for both procedure groups.
Chan et al. [9]	2023	- A dual minimally invasive transforaminal lumbar interbody fusion and minimally invasive lumbar decompression were performed on an 87-year-old patient using spinal anesthesia in conjunction with a liposomal bupivacaine erector spinae block. - The patient tolerated the procedure well with no postoperative complications reported.
Patel et al. [45]	2024	- A systematic review of 17 randomized controlled trails revealed thoracolumbar interfascial plane blocks in conjunction with general anesthesia decreased postoperative pain when a modified injection including anesthetics is injected between the longissimus and iliocostalis.

**Table 2:** Operative stages and goals for awake surgery for the spine.

	Protocol	Medications	Goals
Preoperative Stage	Administration of preoperative analgesics	Methocarbamol, Gabapentin, Acetaminophen	Initial administration to minimize postoperative pain
Intraoperative Stage	Begins with a single-injection administration of spinal anesthesia to the intradural space. Patients may be given a second injection intraoperatively if they complain of discomfort.	Bupivacaine (most widely used) or lidocaine, mild sedation maintained via midazolam	Keep the patient in a semi-conscious, mildly sedated state that prevents limb rigidity but avoids the need for intubation
Postoperative Stage	Postoperative care consists of analgesics, physical therapy, and monitoring	Methocarbamol, Gabapentin, Acetaminophen	Begin physical therapy with the goal of having the patient ambulate by postop day 1

## Data Availability

No new data were created or analyzed in this study. Data sharing is not applicable to this article.
